# Triplet versus doublet therapy in patients with metastatic hormone-sensitive prostate cancer

**DOI:** 10.1038/s41598-026-44627-w

**Published:** 2026-03-16

**Authors:** Keita Hayakawa, Takashi Ueda, Masahiro Iehara, Yusuke Gabata, Junki Murashita, Hikaru Takahashi, Yumiko Saito, Masatsugu Miyashita, Yasuhiro Yamada, Takumi Shiraishi, Atsuko Fujihara, Masayoshi Okumi, Fumiya Hongo, Osamu Ukimura

**Affiliations:** 1 Department of Urology, Kyoto Prefectural University of Medicine, Kyoto-City, 602- 8566 Kyoto Japan; 2 Department of Urology, Graduate School of Medical Science, Kyoto Prefectural University of Medicine, Kyoto, 602-8566 Japan

**Keywords:** Docetaxel, Darolutamide, Abiraterone acetate, Apalutamide, Enzalutamide, Metastatic hormone-sensitive prostate cancer, Cancer, Oncology

## Abstract

First-line treatment options for patients with metastatic hormone-sensitive prostate cancer (HSPC) are divided into two groups: androgen receptor signaling inhibitor (ARSI)-based doublet therapy and triplet therapy (which includes chemotherapy in addition to doublet therapy). However, differences in therapeutic efficacy between the two groups remain unclear. The aim of the study was to perform a comparative analysis between the two groups and to identify predictors of treatment response. We retrospectively recruited 500 patients with mHSPC treated with doublet or triplet therapy from our hospital and affiliated hospitals between 2013 and 2025. The cohort of patients with mHSPC treated with doublet therapy received ABI, ENZ, or APA in combination with a luteinizing hormone-releasing hormone analog, such as androgen deprivation therapy (ADT). The cohort of those treated with triplet therapy received darolutamide plus ADT and docetaxel. Cox proportional hazards regression analysis was performed to identify prognostic factors of overall survival in patients with mHSPC. Propensity score matching was used to adjust the clinical background of patients. The outcome (prostate-specific antigen-progression-free survival, second progression-free survival, and overall survival (OS)) of triplet therapy was significantly better than that of doublet therapy in matched high-risk patients with mHSPC. Univariate and multivariate analyses of OS in matched patients with high-risk mHSPC treated with triplet or doublet therapy suggested that treatment choice (triplet or doublet), pretreatment LDH levels, and presence of Gleason pattern 5 influenced OS in patients with high-risk mHSPC. Subgroup analysis suggested that LDH levels and the presence of Gleason pattern 5 may be predictive factors of the treatment of patients with mHSPC. Our results showed that triplet therapy achieved a better outcome than doublet therapy in patients with mHSPC.

**Supplementary Information:**

The online version contains supplementary material available at 10.1038/s41598-026-44627-w.

## Introduction

A variety of treatment options are available for patients with metastatic hormone-sensitive prostate cancer (mHSPC). In the National Comprehensive Cancer Network (NCCN) guidelines, first-line treatment options for patients with mHSPC are divided into two groups: androgen receptor signaling inhibitor (ARSI)-based doublet therapy and triplet therapy^1^. ARSI-based doublet therapy is a combined therapy of androgen deprivation therapy (ADT) and ARSI: abiraterone acetate plus prednisolone (ABI), enzalutamide (ENZ), and apalutamide (APA). The superiority of doublet therapy to ADT for patients with mHSPC has been demonstrated in a clinical study^2–4^. In triplet therapy, patients with mHSPC are administered docetaxel, a cytotoxic chemotherapeutic agent, in addition to the drugs of doublet therapy. Although triplet therapy is presumed to be superior to doublet therapy from the amount of medication, the adverse events (AEs) of docetaxel should be considered. Although some reports of network meta-analysis have suggested that triplet therapy may be superior to doublet therapy in overall survival (OS) of patients with mHSPC, no direct comparison between triplet and doublet therapies has been reported^5–7^.

Several studies have reported the clinical parameters that affect the efficacy of treatment of patients with mHSPC^8,9^. We previously reported that the histological presence of Gleason pattern 5 (GS5) in the primary tumor may affect the efficacy of ARSI-based doublet therapy of patients with mHSPC. There are few reports on the clinical factors affecting the efficacy of triplet therapy in patients with mHSPC. Previously, we reported a prognostic model for patients with mHSPC treated with ABI using clinical factors including lactate dehydrogenase (LDH) and primary GS5^10,11^.

The purpose of this study was to compare the treatment outcome (prostate-specific antigen-progression-free survival (PSA-PFS), second progression-free survival (PFS2), and OS) between patients treated with triplet and those treated with ARSI-based doublet therapy and to identify the clinical parameters affecting their efficacy.

## Materials and methods

### Patients and treatments

We retrospectively recruited 500 patients with mHSPC treated with doublet or triplet therapy from our hospital and affiliated hospitals between December 2013 and August 2025. The cohort of patients with mHSPC treated with doublet therapy received ABI (1000 mg/day) plus prednisolone (5 mg/day) or ENZ (160 mg/day) or APA (240 mg/day) in combination with a luteinizing hormone-releasing hormone analog such as ADT. The cohort of those treated with triplet therapy received darolutamide (1200 mg/day) plus ADT and docetaxel (75 mg/m^2^ every 3 weeks for six cycles). Bone metastasis was assessed by bone scintigraphy. Visceral and lymph node metastases were assessed by computed tomography (CT). High-risk mHSPC was defined according to the LATITUDE risk stratification (patients with mHSPC and at least two of the following factors: Gleason score (GS) at the primary lesion ≥ 8, ≥3 in bone lesions, or presence of visceral metastasis). Drug selection and dose adjustments were made at the physicians’ discretion. Cutoffs of PSA and LDH were defined as 4.0 ng/mL and 220 IU/L, respectively.

We obtained approval for this study from the Institutional Review Board (IRB) of Kyoto Prefectural University of Medicine. This study complied with the Declaration of Helsinki. The requirement for written informed consent was waived, considering the retrospective nature of the study. Opt-out information was provided to the patients on the website of our hospital and affiliated hospitals.

### Statistical analysis

Chi-square and Wilcoxon rank‐sum tests were used to compare the two groups. Kaplan–Meier analysis, along with the log‐rank test, was employed to estimate the differences in time events between the two groups. Cox proportional hazards models were used to investigate factors associated with PFS. Propensity score matching was used to adjust for clinical background differences between groups. Age at diagnosis in years, performance status, pretreatment PSA and LDH levels, Gleason pattern 5, presence of bone, visceral, and lymph node metastasis, and observation period were used in the propensity score matching. We used SAS JMP, Version 17, for all statistical analyses with a significance threshold set at *P* < 0.05.

## Result

### Clinical characteristics of patients with mHSPC treated with doublet and triplet therapy

The clinical characteristics of the overall cohort in this study are shown in Table 1. A total of 424 patients with mHSPC were treated with doublet therapy (doublet cohort) (180 patients with ABI, 113 patients with APA, and 131 patients with ENZ), and 76 patients with mHSPC were treated with triplet therapy (triplet cohort). The observation period of the triplet cohort was significantly shorter than that of the doublet cohort (*P* < 0.0001). The average age of the triplet cohort was significantly younger than that of the doublet cohort (*P* < 0.0001). Detailed data on sequential therapy following PSA progression are provided in Supplementary Table S1.

**Table 1:** Characteristics of the entire cohort.

Hormone therapy	Triplet (*n* = 76)	Doublet (*n* = 424)			*p*-Value (Triplet vs. Doublet)
		ABI (*n* = 180)	APA (*n* = 113)	ENZ (*n* = 131)	
Median age at diagnosis years (range)	72 (49–87)	74 (53–94)	77 (56–93)	76 (54–89)	< 0.0001
Performance status (ECOG) ≧ 1, n (%)	13 (17.3)	54 (30.9)	32 (28.8)	47 (35.9)	0.0082
Median pretreatment PSA level (ng/mL)	195(0.76–12978)	436 (2.324–24201)	109.508 (0.307–8756)	112.4 (2.58–8413)	0.0451
Median pretreatment LDH (U/L)	195 (100–3456)	198 (39.3–2405)	189 (90–441)	191.5 (14.5–874)	0.0371
Gleason pattern 5, n (%)	49 (64.4)	122 (68.9)	63 (55.7)	71 (54.1)	0.3776
High risk of LATITUDE criteria, n (%)	63 (82.9)	172 (96.1)	68 (60.2)	67 (51.5)	0.0393
Presence of bone metastasis, n (%)	65 (85.5)	165 (92.1)	96 (84.9)	111 (84.7)	0.5636
Presence of visceral metastasis, n (%)	16 (21.0)	50 (27.9)	25 (22.3)	22 (16.9)	0.7015
Presence of lymph node metastasis, n (%)	52 (69.3)	141 (78.7)	69 (61.0)	82 (62.5)	0.8475
Median observation period month (range)	9 (3–28)	34 (3–82)	21 (3–60)	18.5 (3–57)	< 0.0001

### AEs and PSA kinetics of doublet and triplet therapies for patients with mHSPC

AEs occurred in patients with mHSPC treated with doublet and triplet therapies are shown in Supplementary Table S2. Each AE in patients was graded according to the Common Terminology Criteria for Adverse Events (CTCAE) version 5. The details of AEs for each treatment are shown in Supplementary Table S3. In the triplet therapy group, 67 of 76 patients (88.2%) experienced some form of AE, whereas in the doublet therapy group, 70 of 424 patients (16.5%) had AEs. In the triplet therapy group, grade 3 or grade 4 neutropenia was observed in 45 patients, and febrile neutropenia was observed in 20 patients. Forty patients were able to complete all six cycles of docetaxel. Although both doublet and triplet therapies achieved a reduction of ≥ 90% from baseline PSA levels (≥ 90% PSA decline; Supplementary Table S4), grade 3 or higher AEs were more frequent in 50 patients in the triplet therapy group (50 patients: 65.8%) than in the doublet therapy group (16 patients: 3.8%).

### Comparison of outcome (PSA-PFS, PFS2, and OS) in matched patients with high-risk mHSPC treated with doublet and triplet therapies

**Fig. 1:**
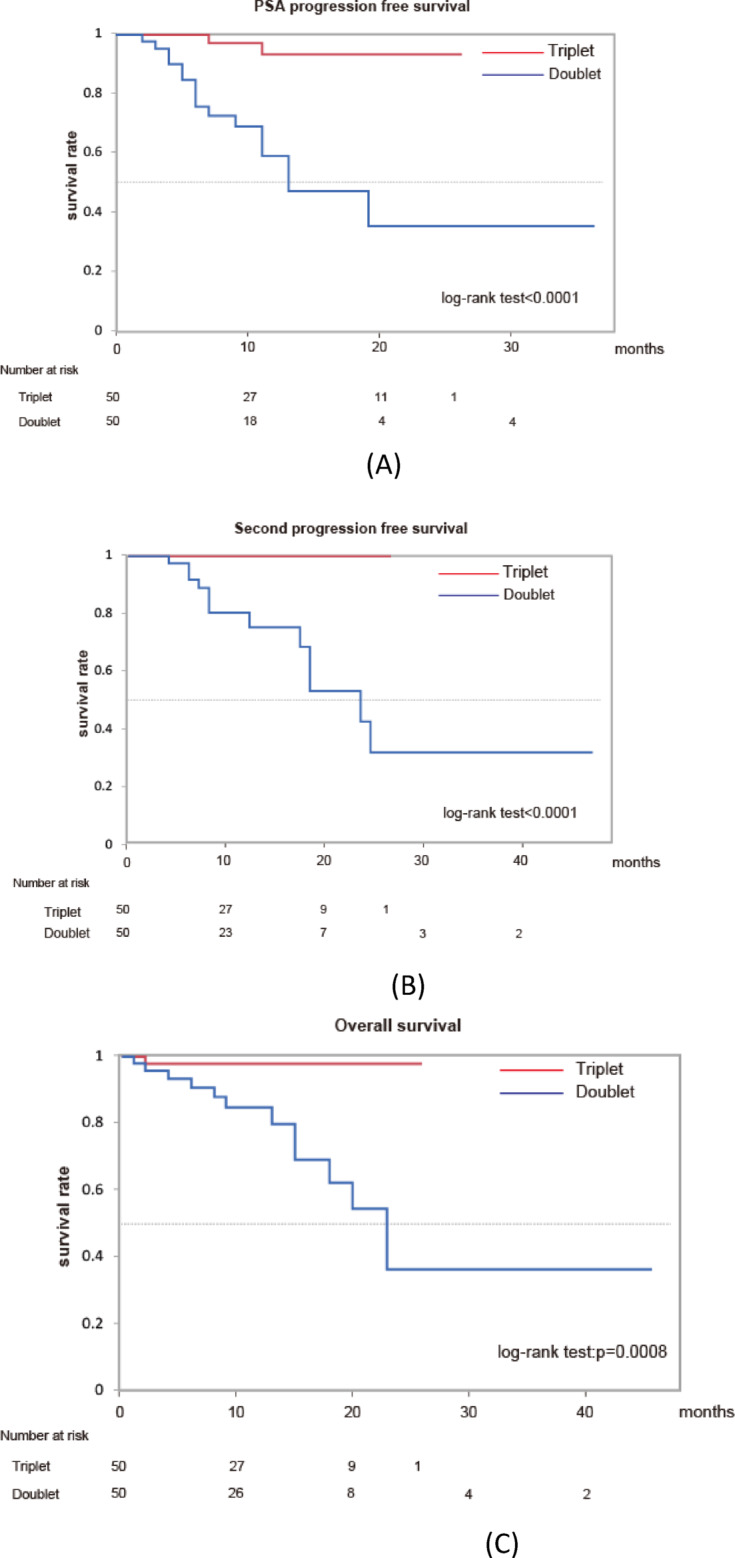
Kaplan–Meier estimates of PSA progression-free survival (**A**), second progression-free survival (**B**), and overall survival (C) in matched patients with high-risk mHSPC (triplet (*n* = 50) vs. doublet (ABI = 26, APA = 8, ENZ = 16)).

Although ABI is indicated only for patients with high-risk mHSPC, triplet and other doublet (APA or ENZ) therapy is indicated for all patients with mHSPC regardless of LATTITUDE risk stratification. We extracted patients with high-risk mHSPC from the overall cohort to compare the outcome between triplet and doublet therapies under the same conditions. Propensity score matching was used to adjust the difference in backgrounds between the triplet and doublet cohorts (Supplementary Table S5). The outcome (PSA-PFS, PFS2, and OS) of triplet therapy was significantly better than that of doublet therapy in matched patients with high-risk mHSPC (Fig. 1). In addition, the median OS was not reached in the triplet group and was 22 months in the doublet group.

### Comparison of outcome (PSA-PFS, PFS2, and OS) in matched patients with mHSPC treated with doublet (ENZ or APA) and triplet therapy

**Fig. 2:**
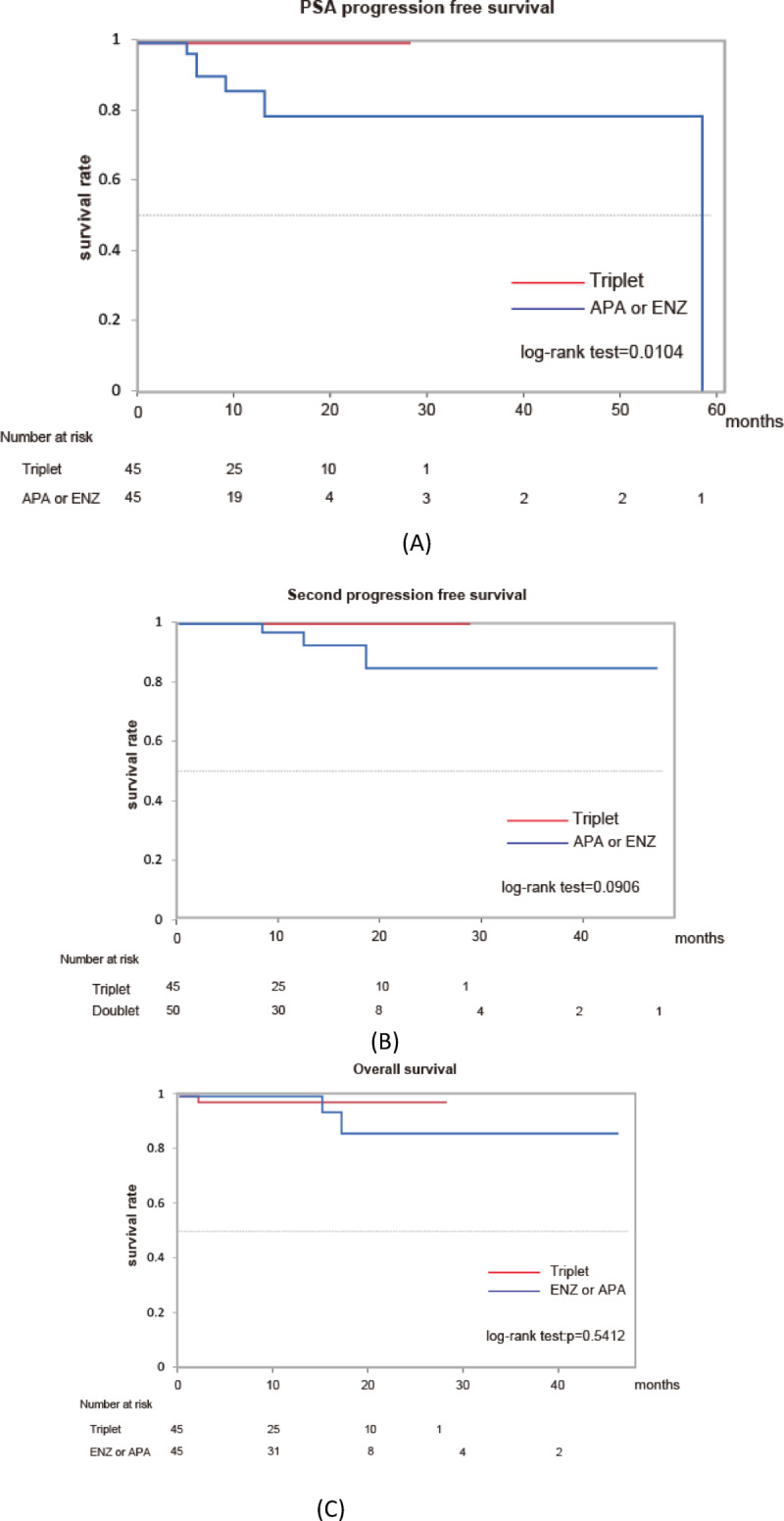
Kaplan–Meier estimates of PSA progression-free survival (**A**), second progression-free survival (**B**), and overall survival (**C**) in matched patients with mHSPC (triplet (*n* = 48) vs. doublet (APA = 20, ENZ = 28)).

Among matched patients with high-risk mHSPC, comparisons among the triplet, ABI, APA, and ENZ groups revealed that the ABI group had poorer outcomes across all endpoints—PSA-PFS, PFS2, and OS (Supplementary Figure S1). In addition, previously, we suggested that ENZ and APA may be superior to ABI in the treatment outcome of patients with mHSPC, especially with Gleason pattern 5 at the primary site^11^. Next, we compared the outcome of triplet therapy with that of doublet therapy, except for ABI in patients with mHSPC. We adjusted the difference in background, including LATTITUDE risk stratification between triplet therapy and the ENZ or APA cohort (Supplementary Table S6). Although PSA-PFS of patients with mHSPC treated with triplet therapy was significantly longer than that of those treated with APA or ENZ, no significant difference was observed in PFS2 or OS between the two groups (Fig. 2).

### Subgroup analysis of patients with mHSPC treated with doublet or triplet therapy based on LDH levels

**Fig. 3:**
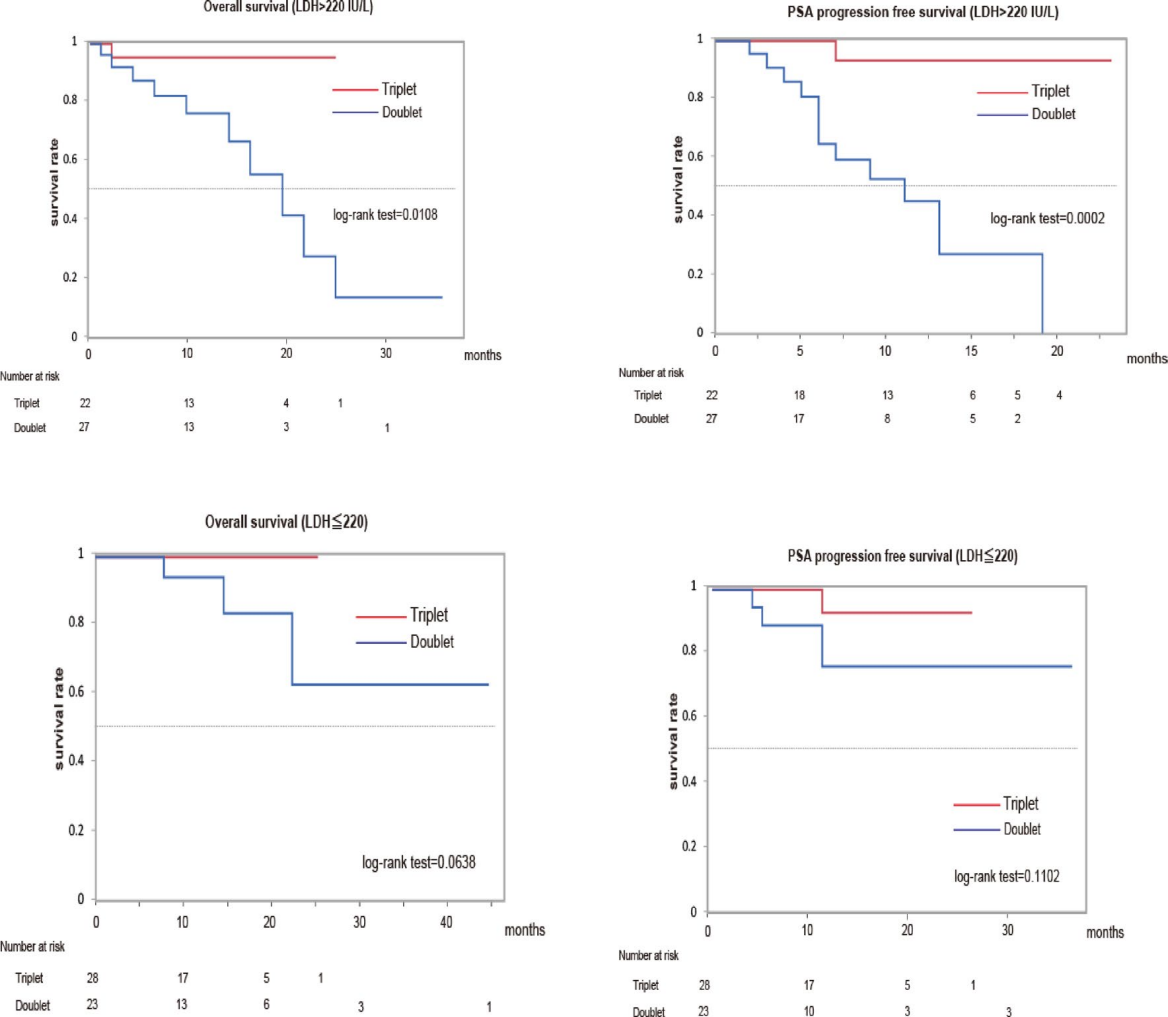
Kaplan–Meier estimates of PSA progression-free survival and overall survival in matched patients with high-risk mHSPC and high (> 220) and low (< 220) LDH levels.

Univariate and multivariate analyses of OS in patients with high-risk mHSPC treated with triplet or doublet therapy suggested that ECOG PS, pretreatment LDH levels, and presence of Gleason pattern 5 might influence OS in patients with high-risk mHSPC (Table 2). From this result, we hypothesized that pretreatment LDH levels and the presence of Gleason pattern 5 at the primary site may be clinical parameters affecting the efficacy of doublet or triplet therapy in patients with mHSPC. To confirm the hypothesis, we performed subgroup analysis by dividing the two groups according to pretreatment LDH levels and the presence of Gleason pattern 5. Although PSA-PFS and OS of patients with mHSPC treated with triplet therapy were significantly longer than those treated with doublet therapy in the subgroup with high LDH levels (> 220 IU/L), no significant difference was observed between triplet and doublet therapies in the subgroup with low LDH levels (≦ 220 IU/L) (Fig. 3). In addition, PSA-PFS and OS of patients with mHSPC treated with triplet therapy were significantly longer than those treated with doublet therapy in the subgroup with the presence of Gleason pattern 5 (Supplementary Figure S2). These results suggested that triplet therapy may be superior to doublet therapy in the outcome of mHSPC with Gleason pattern 5 or high LDH levels.

**Table 2:** Univariate and multivariate analyses for overall survival in patients with high-risk mHSPC.

	Univariate analysis		Multivariable analysis	
	HR (95% CI)	*p*-value	HR (95% CI)	*p*-value
Triplet or doublet	0.75 (0.17–3.20)	0.6948		
Performance status (ECOG)	2.03 (1.18–3.50)	0.010	2.12 (1.19–3.79)	0.0108-
Pretreatment PSA level	1.08 (0.59–1.99)	0.7923	-	-
Presence of GS5	3.68 (1.67–8.15)	0.0013	3.67 (1.40 − 9.63)	0.0083
Pretreatment LDH	3.71 (2.17–6.38)	< 0.0001	2.57 (1.42–4.63)	0.0018
Presence of bone metastasis	2.39 (0.58–9.83)	0.2258	-	-
Presence of visceral metastasis	0.83 (0.47–1.48)	0.5350	-	-
Presence of lymph node metastasis	0.99 (0.52–1.84)	0.9674	-	-

## Discussion

The ARASENS trial demonstrated the superiority in treatment outcome of patients with mHSPC treated with darolutamide-based triplet therapy to those treated with placebo and docetaxel^12^. However, the efficacy of adding docetaxel to ARSI-based doublet therapy remains unclear. In the present study, we retrospectively compared the outcome (PSA-PFS, PFS2, and OS) between patients with mHSPC treated with ARSI-based doublet therapy (ABI, APA, and ENZ) and those treated with triplet therapy.

In this study, propensity score matching was used to adjust for baseline characteristics. Among matched patients with high-risk mHSPC, the triplet group showed significantly better outcomes than the doublet group in terms of PSA-PFS, PFS2, and OS. However, when comparing the triplet group with each of the ABI, APA, and ENZ groups individually, the ABI group showed poorer outcomes across all endpoints. As previously reported, real-world studies comparing ARSIs in patients with mHSPC have indicated that the efficacy of ABI may be relatively modest^13^. Considering that ABI has a different mechanism of action from APA and ENZ, we performed a comparative analysis between the triplet group and the combined APA/ENZ group. The triplet group demonstrated a favorable trend in PSA-PFS, PFS2, and OS; however, a statistically significant difference was observed only for PSA-PFS. Although the matching included observation period, the triplet group had a relatively short follow-up duration, and particular caution is warranted when interpreting the evaluation of OS. Even after excluding the ABI group, the triplet group showed better PSA-PFS outcomes, suggesting that triplet therapy is an effective treatment option. Nevertheless, extending the observation period remains an important issue for future studies.

We found that the outcome of patients with mHSPC treated with triplet therapy was significantly better than those treated with ARSI-based doublet therapy. Although PSA-PFS and OS of patients with mHSPC treated with triplet therapy were significantly longer than of those treated with doublet therapy in the subgroup with high LDH levels (> 220 IU/L), no significant difference was observed between triplet and doublet therapies in the subgroup with low LDH levels (≦ 220 IU/L). The same results were also observed when classified according to the presence of Gleason pattern 5. These results suggested that triplet therapy may achieve better outcomes than ARSI-based doublet therapy in patients with mHSPC and high LDH levels or Gleason pattern 5.

Although triplet therapy achieved a high PSA responsive rate (67%), it also caused a high incidence rate of AEs (AE > G3: 70.5%) in the ARASENS trial^14^. The most common AEs in triplet therapy were neutropenia in the ARASENS trial. The recovery time of patients with mHSPC treated with triplet therapy was significantly prolonged in patients aged over 79^15^. As expected, the incidence rate of AEs (> G3) in patients with mHSPC treated with triplet therapy was significantly higher than in those treated with doublet therapy in the present study. To tailor individual treatment strategies for patients with mHSPC in clinical practice, both the outcome and AEs of triplet therapy should be considered. Older patients with mHSPC (≧ 80 years old) with low LDH levels (≦ 220 IU/L) or without Gleason pattern 5 may be suitable for doublet therapy.

In Japanese health insurance treatments, triplet therapy for patients with mHSPC means a darolutamide-based regimen, and doublet therapy corresponds to an ABI, APA, or ENZ-based regimen. In several countries other than Japan, other ARSIs (ABI, APA, and ENZ) than darolutamide can be administered to patients with mHSPC as triplet therapy, and darolutamide can be administered as doublet therapy. Previous network meta-analysis suggested that the darolutamide-based triplet showed the best outcomes in terms of OS^16^. The differences in response to ARSIs may result from race, as Japanese patients with mHSPC show better response to bicalutamide than other races^17^. The efficacy of these ARSIs in doublet and triplet therapies for Japanese patients with mHSPC should be compared in future studies.

Our study suggested that high LDH levels (> 220 IU/L) or the presence of Gleason pattern 5 in patients with mHSPC was associated with resistance to ARSI-based doublet therapy. A higher level of LDH is associated with poorer outcomes in patients with mHSPC^9,18^. LDH plays a role in tumor proliferation, invasion, and metastasis in colorectal cancers^19^. An immunohistochemical study suggested that LDH overexpression may be associated with poor differentiation. High LDH levels (> 220 IU/L) in patients with mHSPC may be associated with poor differentiation of prostate cancer, which is resistant to androgen blockade therapy, including ARSI-based doublet therapy. Untreated prostate cancer contains a mix of androgen receptor (AR)-dependent and AR-independent cells^20,21^, which forms the rationale for triplet therapy using drugs with different mechanisms of action. In prostate cancers that include Gleason pattern 5, many adverse prognostic gene mutations are present^22^, and a high number of cells resistant to hormonal therapy exist. This is believed to underline the results observed in the present study.

This study has several limitations. Because it was a retrospective study with a small cohort, selection and confounding biases may be present. A prospective study with a larger cohort is needed to exclude these biases. Furthermore, the observation period was very short, especially in patients with mHSPC treated with triplet therapy. Therefore, caution should be exercised when interpreting the evaluation of OS. We plan to perform a study with a longer observation period.

In conclusion, triplet therapy achieved better outcomes (PSA-PFS, PFS2, and OS) than doublet therapy in patients with mHSPC. While caution is needed in interpreting, these results could be useful in clinical practice for treating patients with mHSPC.

## Supplementary Information

Below is the link to the electronic supplementary material.

Supplementary Material 1

## Data Availability

The datasets used and analyzed during this study are available from the corresponding author upon reasonable request.
