# Supplementary material for: Triplet versus doublet therapy in patients with metastatic hormone-sensitive prostate cancer
Source: Sci Rep. 2026 Mar 16;16:13707. doi: 10.1038/s41598-026-44627-w (PMC13125637; doi:10.1038/s41598-026-44627-w)
Supplement: Supplementary file 1 — Supplementary Material 1 [file 41598_2026_44627_MOESM1_ESM.docx]

**Supporting Information**

Supplementary figure S1. Kaplan–Meier estimates of PSA progression-free survival, second progression-free survival, and overall survival in matched patients with high-risk mHSPC (triplet (n=50) vs. ABI=26/ APA=8/ ENZ=16)).

Supplementary Figure S2. Kaplan–Meier estimates of PSA progression-free survival and overall survival in matched patients with high-risk mHSPC and GS5 (left) and without GS5 (right).

Supplementary Table S1. Sequential therapy following PSA progression.

Supplementary Table S2. Adverse events occurring in patients with mHSPC.

Supplementary Table S3. Adverse events in each treatment group.

Supplementary Table S4. PSA kinetics in patients with mHSPC.

Supplementary Table S5. Characteristics of matched patients with high-risk mHSPC (triplet vs. doublet).

Supplementary Table S6. Characteristics of matched patients with mHSPC (triplet vs. APA or ENZ).

**Supplementary figure S1** Kaplan–Meier estimates of PSA progression-free survival, second progression-free survival, and overall survival in matched patients with high-risk mHSPC (triplet (n=50) vs. ABI=26/ APA=8/ ENZ=16)).

**
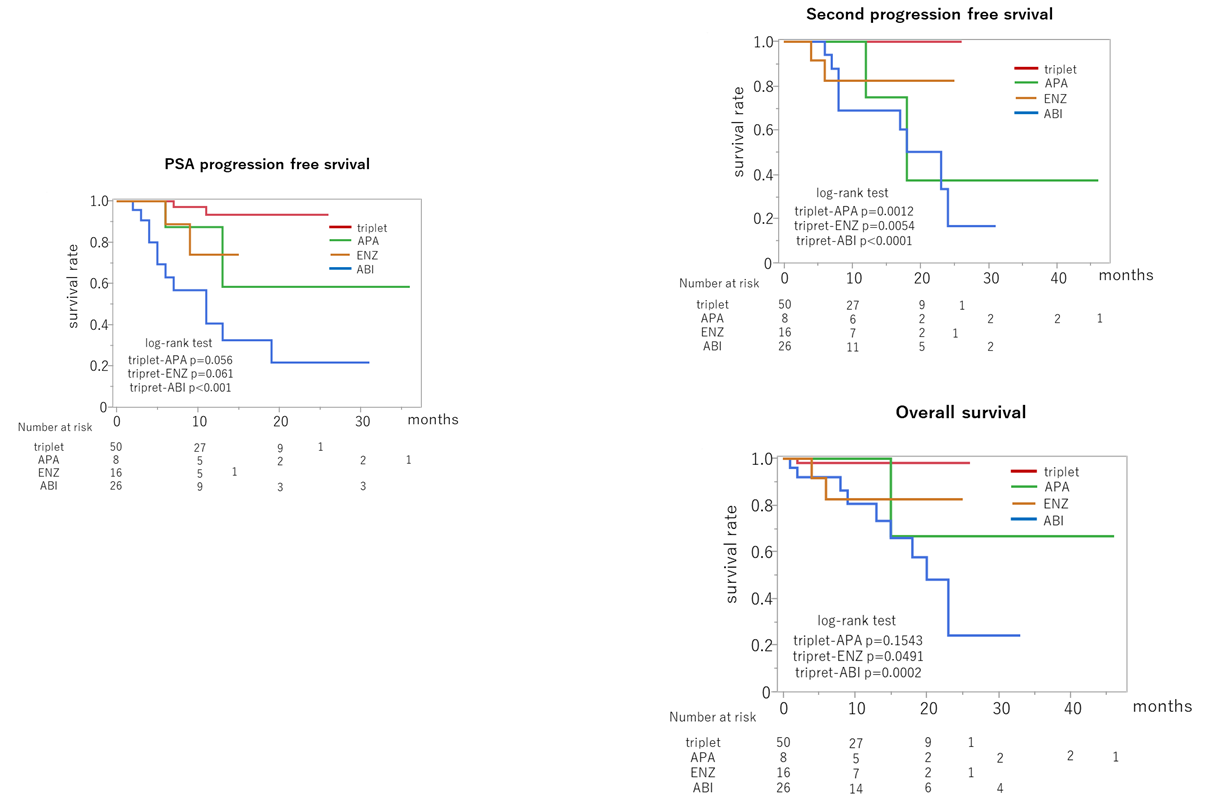
**

**Supplementary figure S2** Kaplan–Meier estimates of PSA progression-free survival and overall survival in matched patients with high-risk mHSPC and GS5 (left) and without GS5 (right).


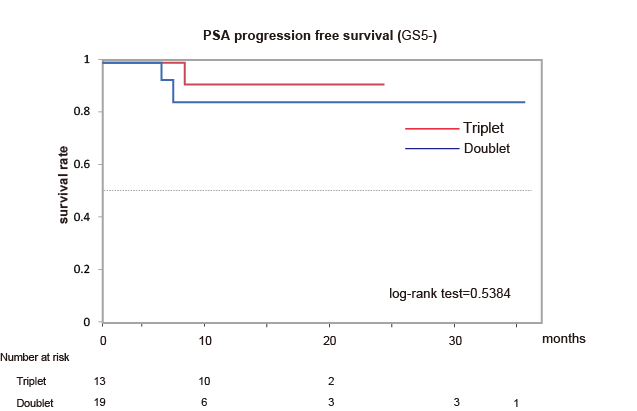


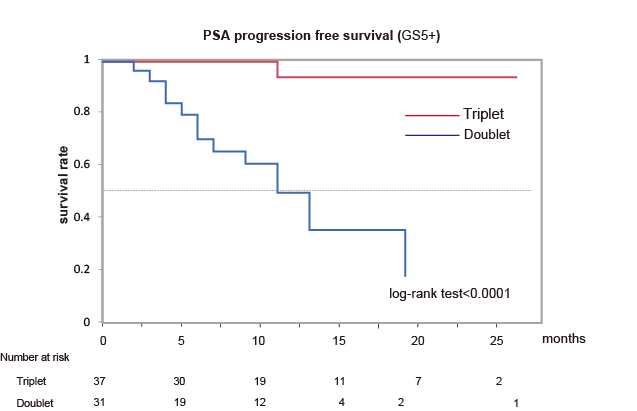


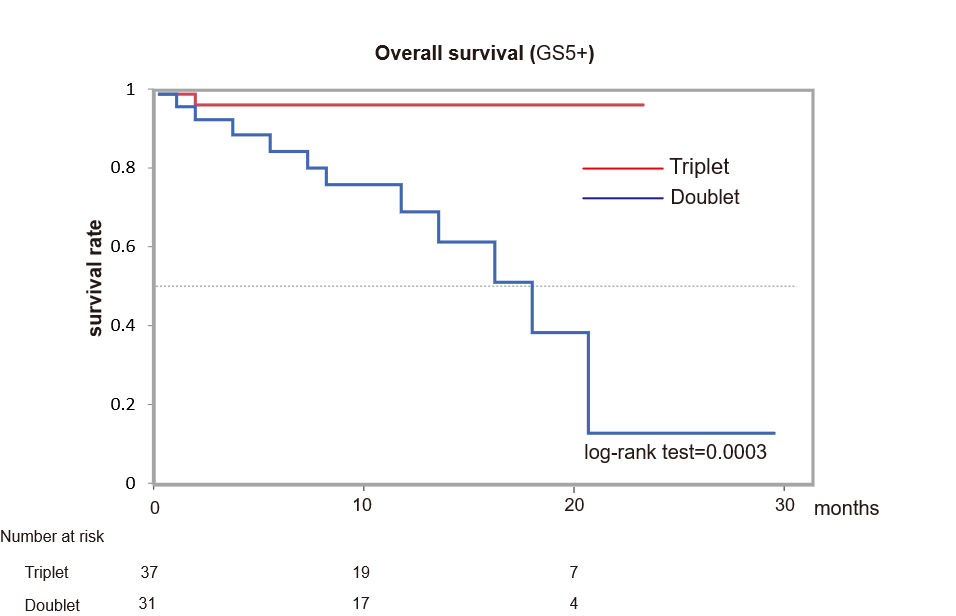

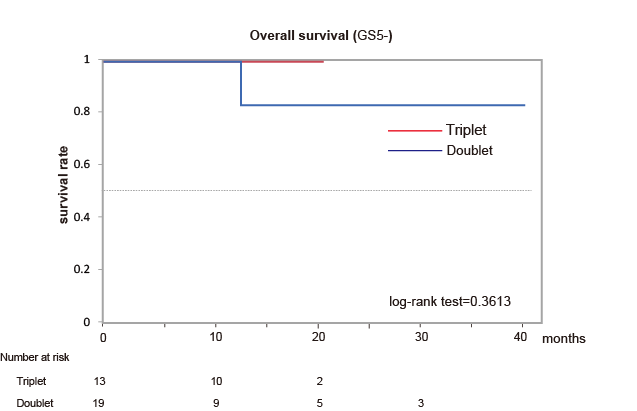


**Supplementary Table S1**. Sequential therapy following PSA progression

| **Agent** | **ABI** | **APA** | **ENZ** | **Triplet** |
| --- | --- | --- | --- | --- |
|  | N =(%) | N =(%) | N =(%) | N =(%) |
| Abiraterone acetate plus prednisone | 0 (0) | 7 (30.4%) | 5 (22.7) | 0 (0) |
| Enzaltamide | 34 (45.3%) | 5 (21.7%) | 0 (0) | 2 (66.7) |
| Docetaxel | 26 (34.5%) | 9 (39.1%) | 10 (45.5) | 0 (0) |
| Apalutamide | 2 (2.7%) | 0 (0) | 1 (4.5) | 0 (0) |
| Olaparib | 0 (0) | 0 (0) | 0 (0) | 1 (33.3) |
| Others | 13 (17.5%) | 2 (8.8%) | 6 (27.3) | 0 (0) |

**Supplementary Table S2**. Adverse events occurring in patients with mHSPC.

| **Highest AE grade** | **Patients n=76 (%)**  **Triplet therapy** | **Patients n=424 (%)**  **Doublet therapy** |
| --- | --- | --- |
| 0 | 9 (11.8) | 354 (83.5) |
| 1 | 7 (9.2) | 37 (8.7) |
| 2 | 10 (13.2) | 17 (4.0) |
| 3 | 10 (13.2) | 15 (3.6) |
| 4 | 40 (52.6) | 1 (0.2) |
| 5 | 0 (0) | 0 (0) |

**Supplementary Table S3.** Adverse events in each treatment group

| Adverse event  (Triplet therapy) | Grade 1 | | Grade 2 | | Grade 3 | | Grade 4 | |
| --- | --- | --- | --- | --- | --- | --- | --- | --- |
|  | No | % | No | % | No | % | No | % |
| Neutropenia | 6 | 7.9 | 4 | 5.2 | 5 | 6.6 | 40 | 52.6 |
| Fatigue | 24 | 31.6 | 3 | 3.9 | 1 | 1.3 |  |  |
| Loss of appetite | 12 | 15.8 | 5 | 6.6 | 1 | 1.3 |  |  |
| Skin disorder | 6 | 7.9 | 2 | 2.6 | 1 | 1.3 |  |  |
| AST/ALT elevation | 7 | 9.2 | 1 | 1.3 | 2 | 2.6 |  |  |
| Electrolyte abnormality | 3 | 3.9 |  |  | 1 | 1.3 |  |  |
| Hypertension | 4 | 5.2 | 1 | 1.3 |  |  |  |  |
| Diarrhea | 5 | 6.6 | 2 | 2.6 |  |  |  |  |
| Constipation | 10 | 13.1 | 2 | 2.6 |  |  |  |  |
| Anemia | 20 | 26.3 | 4 | 5.2 | 4 | 5.2 | 2 | 2.6 |
| Thrombocytopenia | 14 | 18.4 | 3 | 3.9 |  |  |  |  |
| Peripheral neuropathy | 12 | 15.8 | 3 | 3.9 | 1 | 1.3 |  |  |
| Alopecia | 21 | 27.6 | 6 | 7.9 |  |  |  |  |
| Edema | 9 | 11.8 | 11 | 14.5 |  |  |  |  |
| Nail changes | 15 | 19.7 | 2 | 2.6 |  |  |  |  |
| Others | 5 | 6.6 |  |  | 3 | 3.9 |  |  |

**Supplementary Table S4.** PSA kinetics in patients with mHSPC.

|  | ABI | | APA | | ENZ | | Triplet | |
| --- | --- | --- | --- | --- | --- | --- | --- | --- |
|  | No | % | No | % | No | % | No | % |
| PSA decrease of 90% | 172 | 95.6 | 107 | 94.7 | 120 | 91.6 | 70 | 92.1 |

**Supplementary Table S5.** Characteristics of matched patients with high-risk mHSPC (triplet vs. doublet).

| **Hormone therapy** | **Triplet (n = 50)** | **Doublet (n = 50)** | **p-Value** |
| --- | --- | --- | --- |
| Median age at diagnosis years (range) | 73.5 (57-87) | 73 (54-87) | 0.6227 |
| Performance status (ECOG) ≧1, n (%) | 10 (20.0) | 9 (18.0) | 0.9499 |
| Median pretreatment PSA level (ng/mL) | 195 (0.76-12978) | 289.3 (5.54-8756) | 0.5425 |
| Median pretreatment LDH (U/L) | 195 (100-706) | 206 (38.1-2405) | 0.2360 |
| Gleason pattern 5, n (%) | 37 (74.0) | 31 (62.0) | 0.1974 |
| Presence of bone metastasis, n (%) | 47 (94.0) | 48 (96.0) | 0.6453 |
| Presence of visceral metastasis, n (%) | 11 (22.0) | 11 (22.0) | 1.000 |
| Presence of lymph node metastasis, n (%) | 33 (66.0) | 38 (76.0) | 0.2696 |
| Median observation period month (range) | 10.5 (3-26) | 10.0 (3-46) | 0.9413 |

**Supplementary Table S6.** Characteristics of matched patients with mHSPC (triplet vs. APA or ENZ).

| **Hormone therapy** | **Triplet (n = 45)** | **APA(n=21) or ENZ(n=24)** | **p-Value** |
| --- | --- | --- | --- |
| Median age at diagnosis years (range) | 74 (57-85) | 74 (54-86) | 0.9492 |
| Performance status (ECOG) ≧1, n (%) | 11 (24.4) | 8 (17.7) | 0.3969 |
| Median pretreatment PSA level (ng/mL) | 185 (0.76-12978) | 148 (0.307-8756) | 0.8321 |
| Median pretreatment LDH (U/L) | 196 (115-519) | 202 (38.1-441) | 0.8251 |
| Gleason pattern 5, n (%) | 28 (62.2) | 25 (55.5) | 0.5202 |
| High risk of LATITUDE criteria, n (%) | 35 (77.7) | 33 (73.3) | 0.6236 |
| Presence of bone metastasis, n (%) | 39 (86.6) | 36 (80.0) | 0.3948 |
| Presence of visceral metastasis, n (%) | 8 (17.7) | 9 (20.0) | 0.7877 |
| Presence of lymph node metastasis, n (%) | 32 (71.1) | 33 (73.3) | 0.8139 |
| Median observation period month (range) | 11 (3-28) | 11 (3-46) | 0.4921 |
